# Lack of association of baseline 25-hydroxyvitamin D levels with disease severity and mortality in Indian patients hospitalized for COVID-19

**DOI:** 10.1038/s41598-021-85809-y

**Published:** 2021-03-18

**Authors:** Ganesh Jevalikar, Ambrish Mithal, Anshu Singh, Rutuja Sharma, Khalid J. Farooqui, Shama Mahendru, Arun Dewan, Sandeep Budhiraja

**Affiliations:** 1 Institute of Endocrinology and Diabetes, Max Healthcare, Saket, Press Enclave Road, New Delhi, 110017 India; 2 Institute of Internal Medicine, Max Healthcare, Saket, Press Enclave Road, New Delhi, 110017 India

**Keywords:** Infectious diseases, Viral infection

## Abstract

Vitamin D deficiency (VDD) owing to its immunomodulatory effects is believed to influence outcomes in COVID-19. We conducted a prospective, observational study of patients, hospitalized with COVID-19. Serum 25-OHD level < 20 ng/mL was considered VDD. Patients were classified as having mild and severe disease on basis of the WHO ordinal scale for clinical improvement (OSCI). Of the 410 patients recruited, patients with VDD (197,48.2%) were significantly younger and had lesser comorbidities. The levels of PTH were significantly higher in the VDD group (63.5 ± 54.4 vs. 47.5 ± 42.9 pg/mL). The proportion of severe cases (13.2% vs.14.6%), mortality (2% vs. 5.2%), oxygen requirement (34.5% vs.43.4%), ICU admission (14.7% vs.19.8%) was not significantly different between patients with or without VDD. There was no significant correlation between serum 25-OHD levels and inflammatory markers studied. Serum parathormone levels correlated with D-dimer (r 0.117, p- 0.019), ferritin (r 0.132, p-0.010), and LDH (r 0.124, p-0.018). Amongst VDD patients, 128(64.9%) were treated with oral cholecalciferol (median dose of 60,000 IU). The proportion of severe cases, oxygen, or ICU admission was not significantly different in the treated vs. untreated group. In conclusion, serum 25-OHD levels at admission did not correlate with inflammatory markers, clinical outcomes, or mortality in hospitalized COVID-19 patients. Treatment of VDD with cholecalciferol did not make any difference to the outcomes.

## Introduction

Severe acute respiratory syndrome coronavirus 2 (SARS-CoV-2) induced coronavirus disease-19 (COVID-19) pandemic has affected more than 60 million individuals and has claimed more than 1.4 million lives globally since it first broke out in China in November 2019^1^. India has been one of the worst affected countries in terms of the total number of cases (more than 9 million) second only to the United States of America^1^.

Vitamin D is thought to play an important role in respiratory infections. Evidence from observational studies suggests an association between low serum 25-hydroxyvitamin D (25-OHD) level and susceptibility to acute respiratory infections^2^. Several meta-analyses have shown modest protective effects of vitamin D supplementation on respiratory infections^3–5^. The active metabolite of vitamin D, 1,25-dihydroxy-D can directly affect viral replication or immune responses to viral infections including induction of antimicrobial peptides like cathelicidin^6^, regulate immune response by promoting TH2 proliferation and suppression of TH1 proliferation^7^ and modulation of nuclear factor kappa B (NFkB) pathway^8^. In general vitamin D deficiency (VDD) has been observed to lead to dysregulated immune response leading to excessive pro-inflammatory cytokines, implicated in the damage caused by COVID-19^9,10^.

Indirect evidence for the role of vitamin D in COVID -19 is based on the epidemiological studies which reveal higher mortality in countries from the Northern hemisphere which have a higher prevalence of VDD than countries from the Southern hemisphere^11^. Observational studies have also documented a negative correlation between VDD and the total number of COVID-19 cases and COVID-19 associated mortality per million population^12^. The association of low vitamin D with the severity of COVID-19 infection has also been reported^13–15^. However, a small sample size, pre-pandemic 25-OHD levels rather than at the time of infection, and the concomitant presence of other risk factors like obesity and older age make these results difficult to interpret^16^. Hence there is a need for studies to further clarify the role of vitamin D in COVID-19.

Despite being a sunny country, India has a high prevalence of VDD, particularly in urban areas^17^. Interestingly, the case fatality rate (CFR) of COVID-19 in India has also been one of the lowest^18^. In the present prospective observational study, we estimated the prevalence of VDD in consecutive hospitalized Indian patients and studied the association of baseline 25-OHD levels with the severity of COVID-19 infection. The study also provided us an opportunity to see if treatment with cholecalciferol is associated with a change in the outcome of COVID-19.

## Methods

### Study design

This is a prospective, single-center, cross-sectional, observational study carried out at a tertiary care, designated COVID-19 treatment center situated in New Delhi, India. Hospitalized patients were enrolled from July 9, 2020, to August 8, 2020, and were observed till the time of discharge or death while in the hospital. The hospital predominantly caters to the middle and upper socioeconomic class from the National Capital Region of India. The study was approved by the Max Healthcare Ethics Committee, New Delhi, India. A waiver of consent was sought because deidentified patient data was used and the study protocol did not affect the treatment protocol of the patient in any way. The same was approved by the Max Healthcare Ethics Committee. All methods were performed according to the relevant guidelines and regulations.

### Participants

Consecutive patients hospitalized with COVID-19 infection proven by positive nasal and/or nasopharyngeal swab for SARS-CoV-2 by RT-PCR method were included. Patients requiring second hospital admission within the study period were excluded. Asymptomatic patients were generally not hospitalized, except in 17 cases where the patient was either a healthcare worker of home isolation was not possible. A total of 410 patients (including 9 pediatric (< 18 years of age), 17 asymptomatic, 127 females) were included.

### Sample size calculation

The sample size was calculated based on a study comparing parameters of patients requiring ICU admission versus those not requiring ICU admission^13^. A minimum sample size of 319 was calculated to be able to detect a difference of at least 10% in the prevalence of 25-OHD < 20 ng/mL between severe and mild illness with a power of 80% and a significance level of 5%.

### Measurements

Clinical data were collected from the electronic medical records (EMR) including age, sex, presence of comorbidities, presenting symptoms, duration of symptoms, anthropometry, blood pressure, baseline oxygen saturation (SpO_2_), results of laboratory evaluation, and treatment received. All patients were assigned a severity score based on the WHO ordinal scale for clinical improvement (OSCI) (supplementary Table S1)^19^ at hospital admission (baseline) and the highest score during the hospital stay (outcome). Based on the outcome OSCI scores, patients were classified as hospitalized mild disease (3-no oxygen therapy, 4-oxygen by mask or nasal prongs) and hospitalized severe disease (5-non-invasive ventilation or high flow oxygen, 6-intubation and mechanical ventilation, 7-ventilation plus other organ support like inotropes/renal replacement therapy (RRT)/extracorporeal membrane oxygenation (ECMO), 8-death). All patients had a blood sampling done to determine 25-hydroxyvitamin-D (25-OHD) and parathormone (PTH) in addition to the standard COVID-19 protocol which included assessment of inflammatory markers, C-reactive protein (CRP), Interleukin-6 (IL-6), D-dimer, ferritin, lactate dehydrogenase (LDH) and procalcitonin. The level of 25-OHD and PTH was determined using chemiluminescence immune-assay (Beckman Coulter DxI 600 immunoassay system). Vitamin D deficiency was defined by a level of 25-OHD < 20 ng/mL. No change was made in the treatment protocol and the decision of cholecalciferol treatment was as per the treating physician’s decision. For most patients, the treatment was administered as cholecalciferol granules (60,000 units per gram) administered under the supervision of a nurse.

### Outcomes

The primary outcome assessed was proportion of severe cases in VDD versus no VDD. Other outcomes assessed were proportion of cases requiring admission to intensive care unit (ICU), administration of oxygen, inotropic support and renal replacement therapy (RRT). Difference in the mean levels of inflammatory markers was compared. Number of deaths in each of the group was also compared. Finally, outcomes of patients who received cholecalciferol versus those who did not receive cholecalciferol treatment were compared in overall patients and in the subgroup of vitamin D deficient patients.

### Statistical analysis

Statistical analysis was performed using IBM SPSS statistics software version 22.0 (IBM Corp, Armonk NY). Categorical variables were presented as frequency and percentages, whereas continuous variables were described either as mean and standard deviation (SD) or standard error (SE) for mean or median and range. Chi-square test was used to compare differences between categorical variables and the student’s ‘t’ test was used to compare continuous variables. Comparison of continuous variables in more than 2 groups was done using one-way ANOVA test. A ‘p’ value of < 0.05 was considered as significant. Pearson correlation method was used to study the association between 25-OHD, PTH, and outcome severity scores and inflammatory markers.

## Results

### Baseline patient characteristics and overall outcomes of COVID-19 infection

A total of 410 patients (127 females, 9 pediatric, 17 asymptomatic) were included with a median age of 54 years (range 6–92 years). Anthropometry was available for 136 patients and the mean BMI was 27.0.4 ± 4.6 kg/m^2^. At least one comorbid condition was present in 272 (66.3%) patients. Comorbid conditions included diabetes (189, 46.1%), hypertension (164, 40%), hypothyroidism (61, 14.9%), coronary artery disease (CAD) 35 (8.5%), lung or airway disease (24, 5.9%), cancer (11, 2.7%) and chronic kidney disease (CKD) 12 (2.9%). The majority of patients (393, 95.9%) were symptomatic cases with a median symptom duration of 5 days (range 1–20 days). At baseline, a total of 390 (95.1%) patients had mild disease (no oxygen requirement in 318 and low flow oxygen requirement in 72) whereas 20 (4.9%) patients had severe disease (high flow oxygen-18, intubation-1, intubation, and other organ support-1) During the hospital stay, 57 (13.9%) patients had severe outcomes, including mortality in 15 (3.7%), intubation and intubation along with other organ support in 2 (0.5%) each, and high flow oxygen/non-invasive ventilation in 38 (9.3%) patients. A total of 248 (60.5%) patients did not require any supplemental oxygen and 105 (25.6%) required low flow oxygen. Admission to ICU, inotropic support, and renal replacement therapy (RRT) was required 72 (17.6%), 19 (4.6%), and 7 (1.7%) patients each.

### 25-OHD levels in the study population

**Figure 1:**
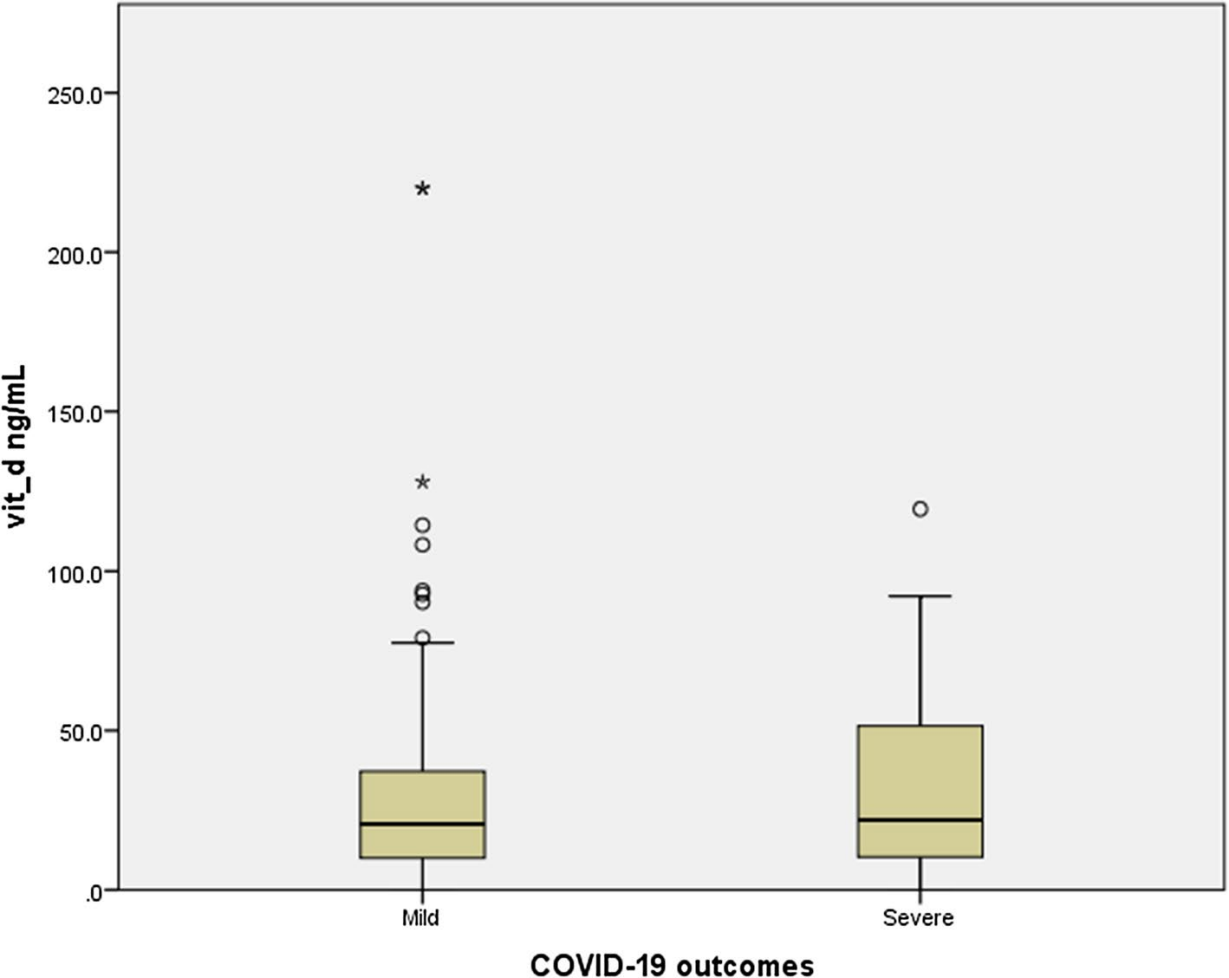
Mean 25-hydroxyvitamin-D levels in mild and severe cases.

A total of 197 (48.2%) patients had VDD (25-OHD < 20 ng/mL), among whom 100 (24.4%) had severe VDD (25-OHD < 10 ng/mL). Levels between 20 and 30 ng/mL, 30–100 and > 100 ng/mL were seen in 67 (16.4%), 139 (34%) and 6 (1.5%) patients respectively. Information about prior vitamin D supplementation was not available. The mean serum levels of 25-OHD in mild vs severe cases (26.3 ± 24.9 vs 31.7 ± 26.8, p-0.165) were not significantly different (Fig. 1).

### Comparison of cases with or without VDD

Table 1 shows the comparison of cases with or without VDD. Patients with VDD were significantly younger and had a lower percentage of comorbidities (including) diabetes and hypertension. The duration of symptoms, percentage of symptomatic cases, and baseline severity including SPO2 were similar in the two groups. There was no difference in clinical outcomes of the two groups with regard to mean outcome OSCI scores, the proportion of severe cases, mortality, requirement of ICU admission, oxygen administration, inotropic support, or RRT. PTH levels were significantly higher and albumin corrected calcium levels were significantly lower in those with VDD. There was no difference between the levels of markers of inflammation (CRP, IL-6, D-dimer, ferritin, and LDH) between the two groups. The findings remained similar after excluding pediatric and asymptomatic cases. The results were the same when patients with severe VDD (25-OHD < 10 ng/mL) were compared to those with 25-OHD > 10 ng/mL and comparison by 4 categories of 25-OHD levels (< 10, 11–20, 21–30 and 30–100 ng/mL).

**Table 1:** Comparison of hospitalized COVID-19 patients with or without vitamin D deficiency (VDD), data presented as number (percentage) for categorical^a^ and mean (SD) for continuous variables^b^.

Parameter	No VDD (n = 212)	VDD (n = 197)	P-value
Age (years)^b^	57.8 (14.7)	46.7 (17.1)	< 0.001
Females^a^	64 (30.2)	63 (32)	0.70
BMI, kg/m^2^ (n = 136)	27.5 (4.8)	27.2 (4.3)	0.67
Systolic BP (mm of Hg)^b^	129.8 (14.8)	128.8 (14.9)	0.48
Diastolic BP (mm of Hg)^b^	78.7 (10)	77.4 (9.7)	0.19
Comorbidities^a^	161 (75.9)	110 (55.8)	< 0.001
Diabetes^a^	111 (52.4)	77 (39.1)	0.01
Hypertension^a^	105 (49.5)	58 (29.4)	< 0.001
CAD^a^	18 (8.5)	16 (8.1)	0.89
CKD^a^	6 (2.8)	6 (3)	0.90
Hypothyroidism^a^	38 (17.9)	23 (11.7)	0.08
Cancer^a^	8 (3.8)	3 (1.5)	0.16
Respiratory disease^a^	14 (6.6)	10 (5.1)	0.51
Symptomatic cases^a^	206 (97.2)	186 (94.4)	0.16
Duration of symptoms^b^ (days)	5.8 (3.3)	5.7 (3.2)	0.67
Baseline SpO_2_ (mm of Hg)^b^	95.6 (3.5)	95.7 (4.6)	0.84
Baseline severity scores^b^	3.3 (0.6)	3.3 (0.6)	0.68
Hospital stay (d)^b^	10 (5.8)	9.2 (5.6)	0.13
Outcome severity score^b^	3.7 (1.2)	3.6 (1)	0.12
Severe cases^a^	31 (14.6)	26 (13.2)	0.78
Oxygen^a^	92 (43.4)	68 (34.5)	0.07
ICU admission^a^	42 (19.8)	29 (14.7)	0.17
RRT^a^	6 (2.8)	1 (0.5)	0.07
Inotropic support^a^	12 (5.7)	7 (3.6)	0.31
Mortality^a^	11 (5.2)	4 (2.0)	0.12
25-OHD (ng/mL)^b^	43.2 (25.8)	9.8 (5)	< 0.001
PTH (pg/mL)^b^	47.5 (42.9)	63.5 (54.4)	0.001
Albumin corrected^b^ calcium (mg/dL)	9.2 (1.2)	9 (0.5)	0.02
CRP (mg/L)^b^	45.1 (56.0)	62.2 (343.7)	0.49
IL-6 (pg/mL)^b^	45.9 (121)	46.3 (113.5)	0.98
LDH (IU/L)^b^	301.1 (123.5)	304 (129.3)	0.83
D-Dimer (ng/mL)^b^	310.8 (722.6)	465.3 (1646.2)	0.22
Ferritin (ng/mL)^b^	311.1 (514.2)	332.7 (618.7)	0.71

When a cut-off value of 30 ng/mL was used to define vitamin D sufficiency, in the group with 25-OHD > 30 ng/mL (n = 145), oxygen administration and ICU admission was required in a significantly higher number of cases compared to those with levels < 30 ng/mL (46.2% vs. 35.2%, p-0.03, 22.8% vs. 14.4%, p-0.04). There was no difference in the mean outcome OSCI scores, the proportion of severe cases, mortality, inotropic support, RRT, or the levels of inflammatory markers. Patients with levels > 30 ng/mL were significantly older (59 ± 14.9 vs. 48.9 ± 16.7 y,p < 0.001) and higher comorbidities (77.2% vs 60.2%, p < 0.001).

To evaluate the impact of confounding factors like age and comorbidities, we conducted a subgroup analysis of 105 elderly patients (age ≥ 65 y), 33 patients with VDD were compared with 72 patients without VDD (Table 2). The proportion of comorbidities was similar in the groups. There was no difference in the clinical outcomes and inflammatory markers.

**Table 2:** Outcomes of elderly hospitalized COVID-19 patients (age ≥ 65 years) with or without VDD, data presented as number (percentage) for categorical^a^ and mean (SD) for continuous variables^b^.

Parameter	No VDD (n = 72)	VDD (n = 33)	P-value
Age^b^	72.9 (6.8)	72.5 (6.7)	0.79
Females^a^	27 (37.5)	14 (42.4)	0.67
Comorbidities^a^	67 (93.1)	29 (87.9)	0.46
BMI (kg/m^2^) (n = 38)^b^	26.5 (3.9)	26.6 (3.7)	0.90
Diabetes^a^	49 (68.1)	20 (60.6)	0.51
Hypertension^a^	46 (63.9)	20 (60.6)	0.83
25-OHD (ng/mL)^b^	47.6 (21.7)	9.2 (4.9)	< 0.001
Severe cases^a^	20 (27.8)	4 (12.1)	0.09
Oxygen administration^a^	46 (63.9)	15 (45.5)	0.09
ICU admission^a^	25 (34.7)	5 (15.2)	0.06
Inotropes^a^	10 (13.9)	2 (6.1)	0.33
RRT^a^	4 (5.6)	0 (0)	0.31
CRP (mg/L)^b^	52.2 (58.5)	61.1 (61.6)	0.49
Ferritin(ng/mL)^b^	351.8 (694.4)	516.7 (1279.9)	0.41
IL-6 (pg/mL)^b^	63.9 (137.4)	77.1 (178.6)	0.70
D-Dimer (ng/mL)^b^	369.9 (424.1)	794.3 (2574.9)	0.35
LDH (IU/L)^b^	318.9 (137.4)	317 (128.3)	0.96

In multivariate analysis (Table 3) neither 25-OHD nor PTH was related to the severity of the disease.

**Table 3:** Multivariate analysis for factors determining the severity.

Parameter	Odds ratio (OR)	95 CI for OR		p-value
		Lower	Upper	
Age	1.05	1.02	1.07	< 0.001
Male sex	2.28	1.1	4.7	0.027
Comorbidities	1.76	0.7	4.4	0.223
25-OHD (ng/mL)	1.00	0.99	1.002	0.870
PTH (ng/mL)	1.00	0.99	1.007	0.666

### Correlation of 25-OHD and PTH with outcome severity scores and inflammatory markers

Pearson correlation between 25OHD showed a weak positive correlation with outcome severity scores and hospital stay (Table 4). There was no significant correlation between 25-OHD level and the inflammatory markers studied. However, there was a positive correlation between PTH levels and D-dimer, ferritin, and LDH levels.

**Table 4:** Correlation of 25-OHD and PTH with outcome severity scores and inflammatory markers in hospitalized COVID-19 patients.

	25-OHD		PTH	
	Correlation coefficient	p-value	Correlation coefficient	p-value
Outcome severity score	0.127**	0.010	0.097	0.052
Hospital stay	0.125*	0.011	0.034	0.491
CRP	− 0.017	0.731	0.035	0.489
IL-6	− 0.023	0.671	0.090	0.098
D-dimer	− 0.074	0.137	0.117*	0.019
Ferritin	− 0.038	0.453	0.132**	0.010
LDH	− 0.012	0.821	0.124*	0.018

**Correlation is significant at the 0.01 level (2-tailed).

*Correlation is significant at the 0.05 level (2-tailed).

### Outcomes of patients treated with cholecalciferol

Cholecalciferol was administered to 128/197 (65%) patients with VDD in a median total dose of 60,000 IU. In the VDD group, cholecalciferol treatment did not change clinical outcomes and was not associated with any difference in the inflammatory markers (Table 5).

**Table 5:** Outcomes of cholecalciferol treatment in hospitalized COVID-19 patients with VDD, data presented as number (percentage) for categorical^a^ and mean (SD) for continuous variables^b^.

Parameter	VDD group		p-value
	Cholecalciferol treatment		
	Yes (n = 128)	No (n = 69)	
Age (years)^b^	45.5 (18.2)	48.8 (14.7)	0.175
Comorbidities^a^	68 (53.1)	42 (60.9)	0.367
Severe cases^a^	14 (10.9)	12 (17.4)	0.269
Mortality^a^	1 (0.8)	3 (4.3)	0.124
ICU admission^a^	16 (12.5)	13 (18.8)	0.292
Oxygen administration^a^	38 (29.7)	30 (43.5)	0.06
CRP (mg/L)^b^	71.2 (427.2)	46.2(64.8)	0.632
IL-6(pg/mL)^b^	41.1 (122.5)	54.6 (97.7)	0.476
D-dimer (ng/mL)^b^	392.7 (1396.3)	602.9 (2042.7)	0.399
Ferritin(ng/mL)^b^	273.5 (355.3)	439.4 (914.5)	0.161
LDH (IU/L)^b^	289.5 (109.8)	333.8 (159.4)	0.065

## Discussion

In this prospective, observational study of 410 Indian patients hospitalized for COVID-19, there was a high prevalence of vitamin D deficiency. However, there was no association between baseline serum 25-OHD level and clinical outcomes of COVID-19 (the proportion of severe cases, mortality, requirement of ICU admission, oxygen, inotropic support, or RRT) as well as the levels of the inflammatory markers. Treatment with cholecalciferol in patients with VDD was not associated with any difference in these outcomes.

Vitamin D has been a matter of intense discussion in the COVID-19 pandemic for its possible role in decreasing the risk of infection as well as affecting the severity of the disease and mortality^20^. India has a high prevalence of VDD^17^, which is also reflected in our study with 48% of the study population being deficient. This percentage was even higher (64.3%) if a 25-OHD cut off of 30 ng/mL was used to define vitamin D sufficiency^21^.

An association between VDD and mortality has been suggested based on epidemiologic evidence of higher mortality in countries with low 25-OHD levels^12^. A study from India correlated historically published data on mean 25-OHD levels with mortality reported from different states, and suggested that mortality may be higher in VDD areas. However this study has major limitations as 25-OHD levels were not measured and most of the historical data used was heterogenous and scant^22^. Several hospital-based studies have reported an association between low 25-OHD and severe/critical COVID-19 disease^14,15,23^, higher rates of ICU admission^13^, higher levels of inflammatory markers^15^and mortality^14,24^. Vitamin D deficiency was shown to be associated with higher morbidity in the elderly^25^ and poor prognosis in patients with respiratory failure^26^. However, most studies are limited by a retrospective design and small sample size. In our study, we did not find any association of serum 25-OHD levels with severe outcomes and higher levels of inflammatory markers. These findings were similar despite using cut-offs of 10 ng/mL, 20 or 30 ng/mL and performing subgroup analysis after removing pediatric or asymptomatic cases. Younger age and lower prevalence of comorbidities in the VDD group could act as a confounding factors in our study but a subgroup analysis of elderly patients with similar prevalence of comorbidities as well as multivariate analysis did not show an association of serum 25-OHD level with severe outcomes. On the other hand, there was a weak positive association of 25-OHD levels with outcome severity scores and hospital stay, and patients with 25-OHD > 30 ng/mL had higher rates of ICU admission and oxygen administration. This finding could be explained by the older age and higher comorbidities in this group. It is noteworthy that one study^27^ did note an association of higher serum 25-OHD level with mortality and another^28^ found longer duration of hospital stay with 25-OHD > 20 ng/mL. One notable difference from other studies was the use of WHO-OSCI scale for defining severity of disease.

A study from Spain^28^ did not find an association of serum 25-OHD level with the severity of the disease but reported significantly high levels of ferritin levels in VDD, which was not seen in our study. Data from a European registry^29^ did not find a relationship between serum 25-OHD levels at onset or after 8 weeks of COVID-19 with disease severity, persistent symptom burden, lung function impairment, ongoing inflammation, or more severe CT abnormalities on follow up. This study reported higher PTH at 8 weeks follow-up in patients who required ICU admission.

Typically, serum PTH level is inversely correlated with serum 25-OHD level. It has been suggested that the PTH level may be a marker of the biological impact of VDD. However, no data is available on PTH levels in the setting of COVID-19. Our study showed that in hospitalized COVID-19 patients, serum PTH level had a weak positive but significant correlation with D-dimer, ferritin, and LDH but not with severity, mortality, or other clinical outcomes.

Although our study was not a randomized controlled trial, we did not find any benefit of cholecalciferol treatment of patients with VDD on outcomes and inflammatory markers. The decision of treatment was based on the 25-OHD levels rather than the severity of the disease in our study and the proportion of cases treated with cholecalciferol was similar across all A recent randomized controlled trial^30^ has reported benefits of short-term high dose cholecalciferol which was shown to be associated with higher number of patients becoming SARS-CoV2 negative with significant decrease in fibrinogen in 7 days. Our findings do not rule out the role of long-term supplementation with vitamin D. Our study, however, does not support benefit of treating VDD with single dose of 60,000 units of cholecalciferol for improving outcomes in hospitalized patients. Our observation needs to be confirmed in adequately powered randomized controlled trials aimed to detect differences in outcomes with vitamin D treatment. It is also possible that daily dosing or higher doses of vitamin D may have a different effect than the treatment used in our study^31^. A recent randomized controlled study, did not show benefit in terms of hospital stay, mortality, ICU admission or need for mechanical ventilation with administration of single large dose of 200,000 units of cholecalciferol in moderate to severely ill hospitalized patients^32^. We also cannot rule out the possible benefits of improving vitamin D status in the general population in regard to reducing the risk of contracting COVID-19. A possibility of type II error of 20% can be there in our conclusion of lack of efficacy of vitamin D in mitigating COVID severity.

The strengths of our study are an appropriate sample size and prospective determination of 25-OHD and PTH in consecutive patients at the time of hospitalization. Apart from it being an observational study, a significant limitation is the lack of information on vitamin D supplementation prior to admission. Obesity is an important contributor to COVID severity, however data on BMI was available only for 136 patients in our study. During hospital stay, cholecalciferol treatment was administered per the decision of the treating physician, and not planned as part of the study, and physician bias in treatment decision and dosing cannot be ruled out.

In conclusion, we did not find any association of VDD with the severity of COVID-19 and mortality in a population with high prevalence of VDD. Serum 25-OHD levels were not associated with levels of inflammatory markers. Treatment of VDD with 60,000 units of cholecalciferol did not seem to offer any benefits with respect to immediate outcomes. While improving vitamin D status of the population to impact bone health remains an important goal for populations with a high prevalence of deficiency, its use in the context of COVID-19 remains questionable.

## Supplementary Information

Supplementary Information

## Data Availability

The datasets generated during and/or analyzed during the current study are available from the corresponding author on reasonable request.
