# Supplementary material for: Lack of association of baseline 25-hydroxyvitamin D levels with disease severity and mortality in Indian patients hospitalized for COVID-19
Source: Sci Rep. 2021 Mar 18;11:6258. doi: 10.1038/s41598-021-85809-y (PMC7973709; doi:10.1038/s41598-021-85809-y)
Supplement: Supplementary file 1 — Supplementary Information [file 41598_2021_85809_MOESM1_ESM.docx]

Supplementary table 1: WHO ordinal scale for clinical improvement (OSCI)

| Patient state | Descriptor | Score |
| --- | --- | --- |
| Uninfected | No clinical or virological evidence of infection | 0 |
| Ambulatory | No limitation of activities | 1 |
|  | Limitation of activities | 2 |
| Hospitalized mild disease | Hospitalized, no oxygen therapy | 3 |
|  | Oxygen by mask or nasal prongs | 4 |
| Hospitalized severe disease | Non-invasive ventilation or high-flow oxygen | 5 |
|  | Intubation and mechanical ventilation | 6 |
|  | Ventilation + additional organ support -pressors, RRT, ECMO | 7 |
| Dead | Death | 8 |

RRT- renal replacement therapy, ECMO- extracorporeal membrane oxygenation
